# Contribution of dermal ethanol absorption during laboratory-controlled high-frequency use of alcohol-based hand rubs followed by glove use

**DOI:** 10.1007/s00204-026-04441-9

**Published:** 2026-05-13

**Authors:** Paul T. J. Scheepers, Kirsten Lassing, Martien H. F. Graumans, Sander H. P. Driessen, Antzelina Gkintsev, Ioanna Konstantinou, Valérie Leermakers, Hilde Onstek, Maxime Sülter, Lotte van den Oord, Chariya van Dolder, Hendrik Jan Jansen, Emilie M. Hardy, Radu Corneliu Duca, Ad Ragas, Frans G. M. Russel, Thomas Rustemeyer

**Affiliations:** 1 Radboud Institute for Biological and Environmental Sciences, Radboud University Nijmegen, Heyendaalseweg 135, 6525 AJ Nijmegen, The Netherlands; 2 Radboudumc Community for Infectious Diseases, Department of Medical Microbiology, Research Institute for Medical Innovation, Radboud University Medical Center, PO Box 1, 6500 HB Nijmegen, The Netherlands; 3 Department of Dermatology, Leiden University Medical Center, Albinusdreef 2, 2333 ZG Leiden, The Netherlands; 4 Department of Pharmacy, Pharmacology and Toxicology, Research Institute for Medical Innovation, Radboud University Medical Center, PO Box 9101, 6500 HB Nijmegen, The Netherlands; 5 Amsterdam University Medical Center, Amsterdam, The Netherlands; 6 Unit Environmental Hygiene and Human Biological Monitoring, Department of Health Protection, Laboratoire national de santé, Dudelange, 3555 Luxembourg; 7 Department Health Protection, Health Directorate, 1445 Strassen, Luxembourg

**Keywords:** Alcohol, Dermal absorption, Skin disinfection, Skin barrier, Occupational health

## Abstract

The safety of using ethanol-based products for hygienic hand disinfection in healthcare workers is controlled by restricting air levels. This raises the question of whether these workers are sufficiently protected against potential dermal absorption. The study aim was to determine the contribution of the dermal route to the total uptake of ethanol. A laboratory-controlled study of 25 sequential alcohol-based-hand-rubs (ABHRs) with subsequent intermediate glove use was conducted in seven healthcare workers. Two exposure conditions were compared: hands and forearms placed inside a laboratory fume hood preventing inhalation (skin-only condition) and the same procedure but outside the fume hood to allow inhalation (native condition). The hand hygiene product contained 84% ethanol and 1% ethanol-d6 added to verify exogenous origin. Inhalation exposure was measured by personal air sampling. Blood was collected at baseline, during exposure and a post-exposure wash-out period and analysed for ethanol and ethanol-d6 by HS-GC-MS. The contribution of dermal uptake was assessed by comparing the area-under-curve (AUC) of blood ethanol concentrations in the skin-only condition with that in the native condition within each study participant. Inhalation exposure in the breathing zone was (mean ± sd): 5.3 ± 3.6 mg/m^3^ in the skin-only condition and 120 ± 7 mg/m^3^ in the native condition. The corresponding AUCs for blood ethanol concentrations were 1400 ± 1100 and 3300 ± 2300 (mg/L)*min (*p* = 0.014), respectively, indicating a contribution from dermal absorption of 43 ± 28% (*N* = 7). For ethanol-d6 these values were 17 ± 4 and 48 ± 17 (mg/L)*min (*p* = 0.064), respectively, with a contribution of dermal uptake of 40 ± 16% (*N* = 3). In conclusion, high-frequency ABHRs lead to significant dermal absorption of ethanol.

**Supplementary Information:**

The online version contains supplementary material available at 10.1007/s00204-026-04441-9.

## Introduction

Ethanol effectively reduces the survival time of pathogens on human skin and is therefore recommended to mitigate infection transmission in patient care, as exemplified during the SARS-CoV-2 pandemic (Hirose et al. 2021; Kramer et al. 2022). However, only few studies have reported on ethanol exposure from hand hygiene practices. Most studies had a focus on compliance issues (Hilt et al. 2020; Kingston et al. 2018; Lebovic et al. 2013). According to a systematic review, the use of alcohol-based hand rubs (ABHRs) monitored by an electronic compliance monitoring system was the highest among nurses in an intensive care unit (ICU), with a reported 95th percentile frequency of 141 ABHR events per shift which corresponds to 15 applications per hour (Boyce et al. 2017). Graham et al. 2005 reported on adverse skin reactions in a university hospital linked to the use of ethanol in hand hygiene. They reported no influence of the frequency or intensity of ABHR use but instead observed an association with irritant contact dermatitis (Graham et al. 2005).

Ethanol is an eye and respiratory tract irritant and neurotoxic at high exposure levels. Consumption of alcoholic beverages is a reproductive hazard, known to cause fetal alcohol syndrome, and is classified by IARC as a confirmed Group 1 human carcinogen (2012). A reduction of the use of alcoholic beverages can prevent cancer (Gapstur et al. 2023). Regarding the use of ethanol as a biocide, the European authority responsible for biocides (ECHA) indicated that ethanol *“may still be approved for the intended biocidal uses*,* if these are considered safe in the light of expected exposure levels”* (ECHA, 2025)).

Exposure to ethanol has been studied by modelling but this was related only to surface disinfection (Anhäuser et al. 2024). The Dutch National Institute for Public Health and the Environment (RIVM) assessed the risk related to occupational ABHR use with exposure modelling adopting the estimated contribution 2% from dermal uptake (Kramer et al., 2007) and found that a high frequency of 32 ABHRs applications per day corresponds to the accepted level for the risk of occupational cancer of 10^− 4^ per year in the Netherlands. Therefore, it was recommended to restrict the number of ABHR uses to not more than 25 times per shift (Hendriks et al. 2021).

On the workplace, exposure to ethanol is usually controlled by air standards only. This raises the question whether workers are sufficiently protected in those exposure scenarios where ethanol is applied on the skin for hand hygiene purposes. In clinical practice, skin disinfection is combined with glove use, causing occlusion of the skin, which may lead to an increased dermal absorption of hydrophilic substances (Meuling et al. 1997; Jones et al. 2003). Here, we conducted a laboratory-controlled study to determine the uptake of ethanol in healthcare workers who regularly apply ABHRs for skin disinfection. Previous studies have measured uptake (Kramer et al. 2007; Hautemanière et al. 2013) but have not attempted to determine the contribution of dermal absorption relative to the total uptake from both inhalation and dermal route. Arndt and co-workers (2014) studied uptake by analysis of ethanol glucuronide excretion in urine. However, they did not perform ethanol air measurements to estimate the contribution from inhalation exposure. Instead, they determined an excretion of 0.09 mg ethanol-glucuronide (EtG) per g of creatinine after applying 32 sequential ABHRs during 8 h with and without local exhaust ventilation (LEV). In the reference condition, when ABHRs were not performed under LEV, the urinary EtG excretion was 0.6 mg/g suggesting an estimated contribution from skin uptake of 15%. The effectiveness of the skin-only condition was not verified by air measurements, making it difficult to interpret these results. Nevertheless, the authors concluded that ethanol is predominantly taken up by inhalation and not via the skin.

The aim of this study was to determine the contribution of dermal absorption to the total ethanol uptake resulting from high-frequency ABHRs. This research question was investigated in healthcare workers who have regular exposure to ethanol-based hand disinfectants and other hand hygiene products that may affect the skin barrier function, such as soap and additional disinfectants.

## Methods

For the current study, healthcare workers who regularly use hygienic hand disinfection were invited to participate in a laboratory-controlled exposure study. It is assumed that they may have a reduced skin barrier function due to the frequent use of ethanol combined with other chemical stressors such as glove use, contact with water (wet work) and with skin disinfectants and other hand hygiene products (Babic et al. 2022). A series of controlled hygienic hand disinfections was performed in a laboratory environment using a commercially available product with a high ethanol content. To attribute the uptake to ethanol from the hand hygiene product, a small defined quantity of deuterated ethanol (ethanol-d6) was added to enable its recovery from blood as a product-specific marker of exogenous exposure. The study design is based on a within-person comparison of two conditions: native and skin-only. The native condition is defined as inhalation combined with dermal uptake and was supported by measurements of the concentration of ethanol in the breathing zone of the study participants. This was the reference comparing the native condition with the skin-only condition. An important addition to previous studies is that we explicitly evaluated glove use under conditions relevant to healthcare practice, as this is expected to modify the dermal absorption of small polar chemicals such as ethanol.

### Recruitment and intake

For this study, ethics approval was obtained in the Netherlands (study protocol no. NL83686.091.23). The study was announced through posters and flyers, and by contacting departments in hospitals where ABHR use was known to be frequent. Healthcare staff interested in participating received written information about the study (see Supplementary material 1) and were subsequently invited to an on-site intake interview with the researchers. After going through the inclusion/exclusion criteria the skin type was determined according to the Fitzpatrick classification (Fitzpatrick et al. 1988). The skin of the hands was assessed by a physician with extended experience in diagnosing hand eczema (LE) to verify that each included study subject had a healthy skin and to examine the medical history with a focus on the history of skin disease and received treatments. This information was used to assess eligibility based on the inclusion and exclusion criteria (see Supplementary material 1).

### Materials and chemicals

For the controlled exposure of participants, Sterillium med (Bode Chemie Hamburg, Germany) was the ABHR-tested disinfectant product, since this is currently one of the most widely used products in Dutch hospital-based healthcare. This product contains 84% of ethanol and 1–2% of isopropyl alcohol. To this disinfectant product, 1% of isotope-labelled ethanol-d6 (99%) (Carl Roth, Karlsruhe, Germany) was added as a marker to confirm the product as the source of blood ethanol. For the study, powder-free nitrile gloves of small, medium and large size were used (Halyard Purezero Life Size Medical Exam Gloves, Halyard, Tilburg, the Netherlands).

For the analysis of blood, standard solutions of ethanol in water for calibration, as well as control whole blood at 304 mg/L were obtained from Medichem Diagnostica (Steinenbronn, Germany). Pure ethanol-D6 was provided by Santa Cruz Biotechnology (Heidelberg, Germany) and prepared as a stock solution of 10 g/L in water. ULC-MS grade water used was purchased from Biosolve (Dieuze, France).

### Preparation for the visits

The study participants were asked to follow a carbohydrate-restricted diet (food and beverage items with not more than 5% of carbohydrates/starch) starting 24 h before their participation. Furthermore, they were asked not to consume any alcoholic beverages on the day before the visits. A journal was kept reporting on any ingredients used when preparing meals at home. This resulted in a well-defined baseline condition and helped to reduce the background of blood ethanol level before and during the exposure and wash-out period.

All study participants were invited to the university early in the morning on the day of the visit. The participants were instructed not to have breakfast and not to use any ethanol-containing stay-on personal care products (PCP) in the morning of the visits. Additionally, they were asked not to use any other hand-applied products, such as creams or sunscreen, because these could interfere with the transepidermal water loss (TEWL) measurements. The first blood sample was collected to determine a pre-exposure blood ethanol baseline (see also paragraph 2.8). After the first blood collection, the study participant was offered a light hydrocarbon-restricted breakfast. Work-related questions were asked as well as questions related to ABHR use, surfactants and skin care products at home. Additionally, a TEWL baseline measurement was done (see below for more details). After that, the participant was taken to the laboratory and following a brief instruction, the ABHRs were applied according to EN1500 (2013) instructions commonly used in hospitals in the Netherlands (Supplementary material 2). Photos of the palm and dorsal side of both hands were taken during each visit.

### Exposure

Controlled exposure to ABHRs were performed in a well-ventilated laboratory facility (with an approximate air exchange rate of 5 times per hour) normally used for laboratory-based courses but not in use for other activities at the time of the study (see Supplementary material 2). For the skin-only condition, the ABHR applications were performed inside a fume hood (Waldner, Wangen im Allgäu, Germany) that had been tested and certified in August 2024. The study participant was instructed to remain seated to ensure a standardised and low physical workload. For this a height-adjustable laboratory chair was used and the study participant remained seated during the entire exposure duration. The equipment used by the researchers was kept in an adjacent fume hood (see photos in Supplementary material 2). The two researchers who carried out the exposure study did not use any alcohol-based personal care products on the day of the exposure. They used a pipette to dispense 3 mL of the disinfection product on the hands of the participant who immediately started to perform the ABHR. During the AHBRs, the hands and forearms remained in the fume hood (see Supplementary material 2). After each ABHR procedure, nitrile gloves were put on, and the study participant was allowed to take a five-minute break moving away from the fume hood but remaining seated. After five minutes, the study participant continued to the next ABHR. This procedure was repeated until the 25th ABHR was completed. This typically took approximately 3 h.

### Wash-out

After the exposure, the study participant was taken to a naturally ventilated office where study participants were allowed to eat and drink. Here a hydrocarbon-restricted lunch was offered. Additional blood samples were collected and TEWL measurements were performed until the last blood sample was collected 4 h after the start of the first ABHR. During the wash-out period, a second air sample was collected (see below for details) to monitor any source of ethanol including the contribution derived from ethanol cleared from the airways of the study participant.

### Inhalation exposure assessment

The exposure to ethanol was measured by personal air sampling using a diffusive sampler type 3 M™ Organic Vapor Monitor 3501+ (3 M Livermore, California, US) placed in the breathing zone on the clothes of the participant. For the exposure (duration of 180 min) and wash-out periods (duration of 60 min), two separate measurements were performed. Air sampling started by opening the sealed packaging and stopped by gastight closing a second bag with a seal provided by the supplier. Air samples were stored at -20 °C until shipment to the laboratory for analysis.

### Room climate measurements

In the laboratory, ambient air temperature, relative humidity and CO_2_ (as a measure of ventilation) were continuously measured using a Rotronic CP11 Indoor Air Quality Meter/Data Logger (Hauppauge, New York, US) and continued in the office during the post-exposure wash-out period.

### Collection of blood

The skin was disinfected with an isopropanol-based product (Cutasept F, Bode Chemie Hamburg, Germany). Because of the repeated blood collection, a Nexiva intravenous catheter 18G green 1.3 × 32 mm (Becton Dickinson, Franklin Lakes, New Jersey, USA) was placed in the fossa cubili. The first blood collection was used to establish a blood ethanol baseline value. The follow-up samples were collected at nine pre-determined time-points after the start of the first ABHR (at t = 10, 20, 30, 45, 60, 75, 90, 120, 150 min during in the exposure phase) and at an additional three timepoints after cessation of exposure during the wash-out phase (at t = 180, 210, 240 min after the start of the 1st ABHR). Blood was collected in 3mL K_2_EDTA Vacutainer tubes (Becton Dickinson), with EDTA used as anticoagulant. After each blood collection, the Nexiva catheter system was flushed with physiological saline solution to ensure sufficient blood flow in the next blood collection. Blood samples were stored at -20 °C until shipment for the laboratory for analysis.

### Analysis of ethanol in air and determination of ethanol/ethanol-d6 in blood

The 3 M 3500 + Organic Vapor Monitor was extracted with 1 mL of carbon disulfide and analysed for ethanol by gas chromatography mass spectrometry (GC-MS) according to the NIOSH method 1400 for Alcohols (National Institute of Occupational Safety and Health, 1994). A reporting limit of 0.2 mg/m^3^ was used.

Analysis of ethanol and ethanol-D6 in blood were performed by headspace-gas chromatography-mass spectrometry (HS-GC-MS) applied on a 10 mL headspace vial containing 200 µL of whole blood transferred after thawing and equilibration at room temperature (22 ± 3 °C). Calibration curves were prepared with aqueous solutions containing ethanol (EtOH) and ethanol-D_6_ (EtOH-D_6_) at concentration levels ranging from 0.1 to 1000 mg/L. The GC-MS system consisted of an Agilent 7890B gas chromatograph equipped with a DB-624 column (30 m, 0.32 mm I.D., 1.8 μm film thickness) coupled to an Agilent 5977B MSD (Agilent Technologies, Diegem, Belgium) operating in SIM mode with electronic impact (EI). Headspace (HS) was performed as follows: sample vial equilibrated at 95 °C for 7 min, injection during 0.5 min with a split 1:6 and the temperatures of the loop and transfer line to the injector, set at 110 °C and 115 °C, respectively. The temperature of the injector and MSD transfer line were set at 250 °C and 280 °C, respectively. The helium carrier gas constant flow was set at 7 mL/min. During the 5.5 min-run of analysis, the temperature of the GC oven was maintained at 40 °C. Table S3.1. (Supplementary material 3) presents the EI-MS parameters used for the selected ion monitoring (SIM) acquisition, identification (see Table 3.1 in Supplementary material 3). Identification and quantification of ethanol and ethanol-D_6_, as well as their limit of quantification (LOQ) in mg/L (LOQ defined as the lowest concentration level with accuracy and precision within 20%). Quality control was ensured with the analysis of a control whole blood sample containing a certified ethanol concentration at 304 mg/L for which the accuracy was determined between 90% and 120% (see Table S3.2. Table 3.2a and 3.2b in in Supplementary material 3).

### Transepidermal water loss (TEWL) measurement

Measurement of TEWL was done in the center of the hand palm as well as centrally on the dorsal side using a VapoMeter (Delfin Technologies, Kuopio, Finland). All TEWL measurements were done in triplicate according to the instructions of the instrument supplier. For the baseline, measurements were repeated until a stable value was obtained to allow the skin to cool and dry following any physical exercise related to travel from home (depending on the mode of transportation by foot, bike, car or public transportation). Following the baseline, measurements of TEWL were taken during 5-min breaks at t = 30, t = 60, t = 120 and the last measurement at the end of the wash-out period at t = 240 min after the start of the exposure.

#### Data analysis

The temperature, relative humidity and CO_2_ data were logged in the memory Rotronic instrument, downloaded and descriptive statistics were generated using the instrument’s software version HG5. The TEWL data were aggregated by calculation of the arithmetic mean and standard deviation for each of the triplicate measurements for both palmar and dorsal side of the hands, separately. Next, for each study participant the relative difference from the baseline was calculated for each of the four points in time for TEWL and 13 points in time for blood ethanol. Blood ethanol and ethanol-d6 concentrations reported as < LOQ (below the limit of quantification) were converted to LOQ/2. This data was then plotted to study the relative development of the blood ethanol concentration within each participant over time for both skin only and native conditions. Area under the curves (AUCs) for TEWL and blood ethanol as well as ethanol-d6 were calculated in Microsoft Excel 2024. The differences between skin-only and native conditions were evaluated by comparison of the area under curve (AUC) of the respective TEWL, blood ethanol and blood ethanol-d6 concentration-time patterns. For each participant AUCs for native and skin-only conditions were tested to find out to what extent the AUC of the native condition was larger than the AUC of the skin-only condition and tested for statistical significance using a one-sided paired Student’s t-test.

## Results

### Study participants

For this study, six females and one male participant were recruited from three different hospitals. The female participants all worked as nurses and the male participant worked as a radiodiagnostic lab technician (Table 1). The participants were employed in different fields of medicine and often working in rotating shifts in departments with high-frequency use of ABHRs. Over the past 6 months typical usage ranged from 25 to 50 ABHRs per shift, with two participants reporting higher ABHR frequencies, ranging 50–100 (Table 1). Three study participants reported the use of water and soap more than 10 times per shift. Only two participants reported the use of skin care products at work and/or at home. The skin health data are presented in Table 2. Four of the study participants had an atopic predisposition based on hay fever. Three participants reported experiencing symptoms of hand eczema within the last 12 months. At the time of the skin assessment, three participants showed signs of xerosis cutis (dry skin), but none had hand eczema.

### Dietary uptake of carbohydrates

Based on the available data the carbohydrate intake was on average (SD) of 47 grams for all participants with an interindividual range from 33 to 66 g (Table S4 - Supplementary material 4). The group averages (SD) during the 24 h pre-exposure period were similar for the skin-only and native condition: 57 and 46 g hydrocarbons, respectively. On the day of the exposure and during the wash-out period, these values were 39 and 44 g of hydrocarbons, respectively. The dietary uptake was not reported for participant 7, and the diary of participant 2 was missing for visits 1 and 2.

### Room climate

The average air temperature, relative humidity and CO_2_ concentration was 20.4 °C, 51% and 420 ppm, respectively, in the laboratory and 22.3 °C, 47% and 443 ppm respectively. A complete overview of the climate conditions during the exposure and wash-out phase for both native and skin-only conditions can be found in Table S5 of Supplementary material 5.

### Air concentrations

Ethanol exposure in the breathing zone of the study participant during the skin-only condition was 5.3 ± 3.6 mg/m^3^ and during the native condition 120 ± 70 mg/m^3^ (*N* = 6). During the wash-out phase, the ethanol concentrations in the office room air were 4.5 ± 2.4 mg/m^3^ following the skin-only condition and 6.8 mg/m^3^ following the native condition. A summary of the data per study participant is presented in Table 3.

**Table 1:** Characteristics of study participants

Study parti-cipant	Gender	Age (y)	BMI (kg/m^2^)	Job title	Department (hospital^c^)	Type of shifts	No. of water/soap uses per shift^b^	No. of ABHRs uses per shift^b^	Use of skin care products at work^b^	Use of skin care products at home^b^
1	F	32	20	Nurse	ICU (B)	d-e-n	<=10	25–50	No	No
2	F	33	24	Nurse	Gastro-enterology (B)	d-e	11–25	51–100	Yes	Yes
3	F	54	35	Nurse	Pediatric ICU (B)	d-e-n	<=10	25–50	No	Yes
4	F	39	25	Nurse	ICU (B)	d-e-n	<=10	25–50	No	No
5	F	26	20	Nurse	Flex^d^ (B)	d-e	<=10	25–25	No	No
6	M	34	27	Radio-diagnostic lab technician	Radiology (C)	d-e-n	> 100	25–50	No	No
7	F	23	24	Nurse	Internal medicine (A)	d-e-n	11–25	51–100	No	No

^a^ d = day, e = evening, n = night; ^b^On average during past 6 months; ^c^A = hospital A, B = hospital B, C = hospital C. ^d^Flex = working at different departments

**Table 2:** Skin health assessment

Skin health assessment		Study participant						
		1	2	3	4	5	6	7
Questions	Did you (as a child) have asthma, hay fever or eczema?	Hay fever	No	Hay fever	Hay fever	No	Hay fever	No
	Do you have a skin anomaly for which you consult a general practitioner or dermatologist. If yes, type of anomaly?	No	No	No	No	No	No	No
	Did you have any of the following complaints about hands or fingers during the past 12 months: red or swollen fingers, itches, desquamation, cracks, blisters or red bumps?	Yes, flaking	No	Yes, cracks	No	No	No	Yes, cracks
	Did you ever use a hormonal creme or any other medicinal creme for dermal anomalies to the hands? If yes, do you still use these and how many times a week?	No	No	No	No	No	No	No
Skin type^a^	Fitzpatrick classification	2	2	2	2	2	3	1
Anomalies	Xerosis cutis	Yes	No	Yes	Yes	No	No	No
	Hand eczema	No	No	No	No	No	No	No
	Any other related complaints or observations regarding the skin of the hands	Mild desquamation lateral palmar side due to friction caused by bouldering	n/a	Interdigital and digi 1 and around cuticles xerosis cutis	Interdigital and digi 1 severe xerosis cutis no erythema	n/a	n/a	n/a

^a^Skin types: Type 1: White skin, always burns, never tans; Type 2: Fair skin, always burns, tans with difficulty; Type 3: Average skin color, sometimes mild burn, tan about average; Type 4: Brown skin, never burns, tans very easily; Type 5: Black skin, heavily pigmented, never burns, tans very easily

### Blood ethanol concentrations

**Fig. 1:**
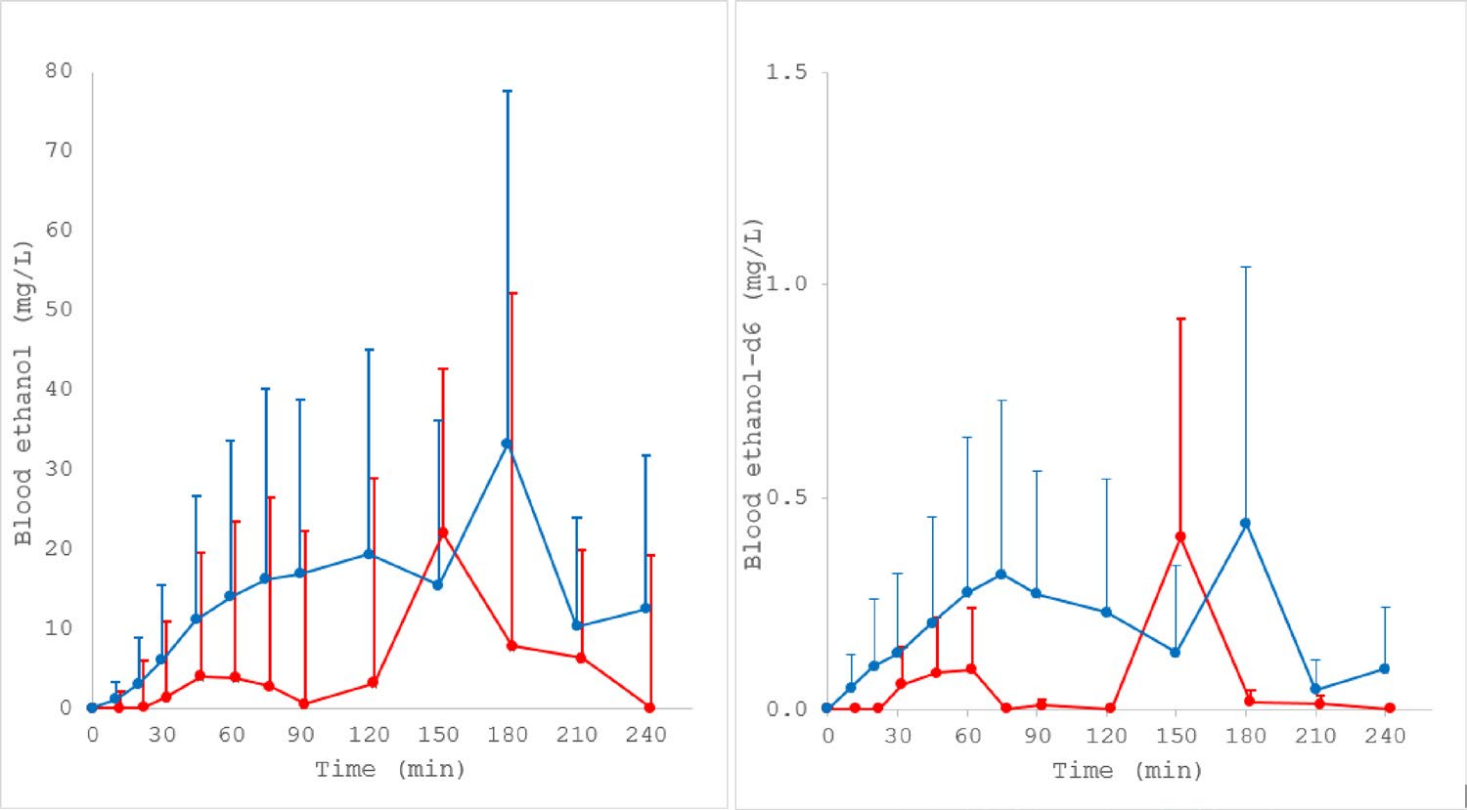
Blood concentrations for ethanol (left) and ethanol d6 (right) following 25 ABHRs. Results are presented for the native condition (blue) and skin-only condition (red) as arithmetic mean with error bars indicating standard error of the mean (AM ± SEM). Paired native and skin-only concentration-time patterns for ethanol and ethanol-d6 of individual study participants can be found in Supplementary material 7

In blood, the baseline levels of ethanol for study participants 1, 4 and 5 ethanol were all below the LOD. For study participants 2, 3 and 6, small amounts of ethanol at 0.06, 0.05 and 0.04 mg/L were detected respectively (all below the LOQ). The concentration time-patterns for the two exposure conditions, based on blood concentrations of ethanol quantified in 7 study participants, are shown in Fig. 1. For ethanol-d6 only data from study participants 1, 4 and 7 could be used because for the other study participants concentrations were below the LOQ for most timepoints. The concentration-time patterns for ethanol and ethanol-d6 are very similar and the concentration of ethanol-d6 is approximately 100 times lower than that of ethanol, consistent with the expected ratio based on the addition of 1% ethanol-d6 to the product already containing 84% of ethanol. Both concentration-time profiles show an immediate increase from t = 0 in the native condition, whereas the skin-only condition exhibits a small delay of approximately 30 min. The highest concentration was reached approx. two hours after the start of the exposure.

Based on these data the average AUCs for blood ethanol were 1400 ± 1100 mg/L*min and 3600 ± 2300 mg/L*min for skin-only and native conditions, respectively (Table 3). These AUCs indicate an average contribution from dermal absorption (expressed as AM ± SD) of 43 ± 18% with a p-value (paired, one sided) of 0.014 based on the measurement of ethanol during the skin-only condition compared to the native condition as a reference. For ethanol-d6 there where sufficient detects > LOQ were available to allow a meaningful calculation of the average AUC for three study participants for both exposure conditions, resulting in an AUC (expressed as AM ± SD) of 17 ± 4 mg/L*min for the skin-only condition and 48 ± 17 mg/L*min for the native condition. This resulted in an average contribution from the dermal absorption of 40 ± 16% with a p-value of 0.064.

### TEWL measurements

We did not observe an impaired barrier function of the skin as TEWL values measured in triplicate were mostly in the normal range (Oestmann et al. 1993; Mayrowitz 2023) except for four somewhat enhanced values (47, 48, 53 and 54 g/m^2^/h) on the palmar side for participant 2 who reported a higher frequency of surfactant (11–25 times per shift) and high frequency ABHR use (50–100 per shift) and also for four values for study participant 6 (54, 60, 64 and 79 g/m^2^/h) who reported a high frequency use of surfactant use of > 100 times per shift (Table 1). Supplementary material 6 provides an overview of the raw TEWL data. For each participant the development of TEWL is presented from the baseline before the start of exposure (at t = 0) and subsequently three measurements during the ABHR series. The last timepoint (t = 240) was from a measurement during the wash-out phase. Each point is the average of a measurement in triplicate relative to the TEWL at baseline. For four study participants (no’s 1, 3, 4 and 6 a concave development over time was observed. For two participants (Arndt et al. 2014, Bessonneau and Thomas 2012) we observed a gradual increase until t = 240 min for the TEWL of the hand palm. For participant 7, TEWL measurements were not available due to a technical problem with the instrument.

**Table 3:** Air concentrations in the breathing zone and blood ethanol levels following 25 ABHRs for the skin-only and native exposure conditions. Air concentrations are expressed as time-weighted average over the exposure period (approx. 3 hours) and subsequent wash-out period in an office environment (approx. 1 h) in µg/m^3^. Blood ethanol is presented as AUC (mg/L*min) to compare skin-only with native condition for the contribution of skin absorption expressed as a percentage of the total uptake (by dermal and inhalation) for both ethanol (*N* = 7) and ethanol-d6 (*N* = 3). Time-concentration plots for individual study participants are provided in Supplementary material 7(S7a–g)

Study partici-pant	Time-weighted average concentration of EtOH in air (µg/m^3^)				AUC of EtOH in blood (mg/L x min)		Estimated contribution of skin to total uptake (skin + inhalation) (%)	AUC EtOH-d6 in blood (mg/L x min)		Estimated contribution of skin to total uptake (skin + inhalation) (%)
	Skin-only condition		Native condition (uptake by skin + inhalation)		Skin-only condition	Native condition (uptake by skin and inhalation)		Skin-only condition	Native condition (uptake by skin and inhalation)	
	During exposure to 25 ABHRs	Until 240 min after start of exposure	During exposure to 25 ABHRs	Until 240 min after start of exposure						
1	11	5	208	11	814	932	**87**	–^b^	–^b^	–^**b**^
2	7	6	63	5	1999	2733	**73**	–^b^	–^b^	–^**b**^
3	6	8	58	4	3380	5091	**66**	22	36	**61**
4	6	5	215	6	530	4669	**11**	–^b^	–^b^	–^**b**^
5	0.8	0.9	37	4	144	1581	**9**	–^b^	–^b^	–^**b**^
6	0.8	2	151	11	2256	8216	**27**	17	72	**24**
7	–^a^	–^a^	–^a^	–^a^	668	2369	**28**	12	36	**34**
**AM ± SD**	**5.3 ± 3.6**	**4.5 ± 2.4**	**122 ± 73**	**6.8 ± 3.0**	1400 ± 1100*	**3600 ± 2300**	**43 ± 28**	**17 ± 4**	**48 ± 17**	**40 ± 16**

^a^No data available; ^b^Not sufficient EtOH-d6 data > LOQ to be able to calculate an AUC; *p < 0.05 in paired Student t-test, comparing the AUC of ethanol in blood in the skin-only condition to the AUC in the native condition.

**Table 4:** TEWL measurements on two sides of the dominant hand during the exposure and wash-out periods normalised to the baseline values expressed as AUC (in kg of water per m^2^ skin surface multiplied by exposure time in hours)

Study participant number	AUC of TEWL (kg/m^2^*h)^b^			
	Palm side of hand		Dorsal side of hand	
	Skin-only	Native	Skin-only	Native
1	507	760	487	554
2	600	610	568	623
3	370	411	455	621
4	469	610	532	594
5	554	529	455	447
6	733	749	722	655
7	–^**a**^	–^**a**^	–^**a**^	–^**a**^
**AM ± SD**	**540 ± 100**	**610 ± 110**	**540 ± 90**	**580 ± 110**

^a^ Not sufficient data to calculate an AUC; ^b^TWEL values can be found in Supplementary material 6

## Discussion

In this study, two standardised conditions of high frequent use of ABHRs were compared within a controlled laboratory setting. The ABHR procedure was performed by participants who routinely apply ABHRs at a high frequency during their normal duty shifts. The primary outcome measure was the AUC of blood ethanol concentrations over a four-hour period. The effect of the skin condition was studied by TEWL as a secondary outcome. In addition to what other studies already provided, the air concentration measured in the breathing zone of the study participant was used as an estimate of inhaled ethanol. These data were combined to quantify the relative contribution of dermal absorption as a fraction of total uptake (inhaled and dermal). The climate and ventilation conditions during the laboratory and office stays were standardised and were similar during the skin-only and native conditions. The most important difference was the contribution of ethanol inhalation exposure that was measured during the skin only condition and amounted to an average of approximately 4.3% of the exposure during the native condition. Assuming that the ABHRs were similarly performed by healthcare workers who routinely use them during their daily work, we consider it appropriate to compare the blood results within each study participant to infer the contribution of dermal absorption relative to total uptake (inhalation and skin).

### Exposure

According to the instructions, the hands should be rubbed with the disinfection product for 30 s until the hands are dry. In practice, this time is often much shorter, and nurses will put on gloves as soon as the skin surface feels sufficiently dry. The study participant indicated that it is not possible to put on gloves when ethanol is still evaporating from the skin surface. In clinical practice, they indicated putting on gloves as soon as the skin ‘felt’ dry. This was the procedure followed during the current study. For the type of product used in this study, the estimated drying time may therefore be shorter than 30 s, even for a product with an ethanol concentration of above 80% (Pendlington et al. 2001; Macinga et al. 2014). The air concentration of 122 ± 73 mg/m^3^ (range 37–215 mg/m^3^) was high compared to conditions studied by Hautemanière and co-workers (2013), who reported a correlation between ABHR use frequency and air concentrations in the breathing zone in a French university hospital. In that study, a frequency of 25 ABHRs corresponds to an air concentration of approx. 30 mg/m^3^. This means that the current lab study (with an air exposure level of 122 ± 73 mg/m^3^**)** does not likely overestimate skin uptake to the total intake as observed in a real-world workplace setting (Hautemanière et al. 2013). When applied in a series with intermediate glove use, as done in this study, the skin is likely to become hydrated as indicated by the relative increase for the TEWL that returned to the baseline in most participants (Table 4 and Supplementary material 6). An increase in the water content of the uncovered skin, combined with the occlusion during glove wearing, may enhance dermal permeation by up to a factor 20, as reported in vitro (Pendlington et al. 2001).

The air concentration during the skin-only exposure condition was 5.3 ± 3.6 mg/m^3^ corresponding to 4.3% of the average concentration during the native exposure condition. The study participant is the most likely source of this background, exhaling < 0.01–0.2 µg/L in his or her own breathing zone and thereby recirculating ethanol. This was particularly relevant during the wash-out period because then the participant was staying in lower ventilated office compared to the laboratory. As can be seen, concentrations measured during the stay in the office (mean 4.5 mg/m^3^ for the skin-only condition and 6.8 mg/m^3^ for the native condition) were similar to the levels observed in the laboratory during the skin-only condition (5.3 mg/m^3^). At the time of the exposure the laboratory was completely empty with all the tabletops cleared and all lab alcohol removed. Some ethanol may have been released when taking off gloves during the 5-min intervals between ABHRs on three occasions during the ABHR series (t = 30, 60, 120 min) during TEWL measurements.

Dietary ethanol intake of the study participants was restricted to 22% of the average carbohydrate intake of 213 g/d (Cosun Nutrition Center, 2024) which resulted in baseline blood ethanol levels below the LOQ at the start of the visit. Only 3 of the 14 baseline levels (participants 3, 4 and 6) showed a small trace of ethanol on one of their visits. This resulted in a well-defined condition with a low baseline of blood ethanol before the exposure started. For both conditions the concentration time-pattern in Fig. 1 showed a gradual increase of the ethanol concentration until a maximum was reached. The concentration was not completely back to baseline for four of the study participants no’s 2, 3, 5 and 7. The difference between the skin-only and native condition was the most remarkable in the first two hours of exposure when the uptake by inhalation increased faster in the native condition compared to the skin-only condition. After that timepoint the person-to-person variability makes it more difficult to compare the native with the skin-only condition.

The small amount of ethanol-d6 added to the disinfectant product used during the ABHRs confirmed the origin of the blood ethanol measurements and indicates that contributions from other exogenous or endogenous ethanol sources were negligible. The concentration-time profiles for ethanol and ethanol-d6 were very similar despite the limited quantifiable ethanol-d6 data in three of the study participants.

Based on the available data we confirmed a higher uptake in the native condition compared to the skin-only condition. This pattern was also reflected in the AUC derived from the more limited ethanol-d6 dataset, although statistical significance was not reached due to considerable interindividual variability. The participant with the highest contribution from dermal absorption (87%) reported to perform bouldering exercise as leisure activity. The hand palms showed some more callus than usual, but the skin was evaluated as healthy (no reason for exclusion). Calluses by itself will not reduce skin barrier function (Pedersen and Jemec 2006). However, an in vitro study by Kikuchi et al. 2020 showed that permeability of the skin to an aqueous solution increases both with the frequency and intensity of rubbing, and concluded that rubbing markedly reduces the barrier function of the stratum corneum (Kikuchi et al. 2020). The participant with the second-highest contribution from skin absorption (72%) reported the most frequent ABHR use at work (50–100 applications per shift), whereas all other participants reported lower use (25–50 applications per shift). The participant with the lowest contribution from skin uptake (9%) had the shortest work history as a nurse (< 2 years).

An increased TEWL is an indicator of decreased skin barrier function (Plum et al. 2020). Especially in healthcare, the use of surfactants and disinfectants may alter the barrier function in the hands (Babiç et al. 2022; Hui-Beckman et al. 2022). Skin barrier has a strong influence on the dermal absorption of hydrophilic compounds with a Log Po/w of < 1 (Levin and Maibach 2005). TEWL is used as a predictor of dermal permeability for both hydrophilic and lipophilic compounds (Tsai et al. 2001) and was reported to increase by a factor two in hydrated compared to dehydrated human skin (Hikima and Maibach 2006). In ex vivo surgical skin immersed in 80% ethanol, the barrier function was disrupted and the stratum corneum thickness was reduced, yet TEWL did not increase (Barthe et al. 2024), suggesting that ethanol in ABHR products may enhance its own dermal absorption through mechanisms other than TEWL. In the current study, the TEWL measured during the skin-only and native conditions were similar (no significant difference). The TEWL baselines for each participant were determined after the first blood had been collected and breakfast had been consumed, on average approximately 1 h after arrival. This procedure resulted in a stable baseline with limited or no significant influence from preceding physical exercise related to mode of commuting (walking or biking). TEWL measurements indicated normal baseline values (average of triplicate TEWL values in g/m^2^/h) ranging from 14.7 to 32.3 on the palmar side and from 7.6 to 15.8 on the dorsal side of the dominant hand. Of the TEWL values measured at 30, 60 and 120 min on both the palmar and dorsal sides after the start of the exposure, the largest increases relative to baseline were observed in participant 2 (64%) and participant 6 (251%) (see Supplementary material 6). For these participants this is consistent with the reported higher frequency of water/soap and ABHR use during their work. TEWL remained elevated relative to baseline and still going up at t = 240 min in the hand palm in study participant 2 and 4 (both skin-only and native conditions) and 5 (native condition, no data available on skin-only condition), at 4 h after the start of the skin-only and native ABHR applications, whereas participants 1, 3 and 6 showed almost complete recovery (see Supplementary material 6). The TEWL for the palmar and dorsal side of the hands were not associated with the % predicted dermal absorption. A similar laboratory-controlled study in humans also reported only a slight increase from baseline (Plum et al. 2020). In vitro studies using a pig skin model indicated that the permeation increased by a factor of 20 (2.19 mg/cm^2^ for occluding vs. 0.10 mg/cm^2^ for non-occluding conditions) (Pendlington et al. 2001). In the current study, the skin was not covered from the start of the exposure, however, regarding the high frequency of ABHR applications and the intermittent use of gloves, occlusion conditions are expected to occur. This is supported by the increase in TEWL from baseline to the 5th ABHR application at t = 30 min (except for study participant 3) (Supplementary material 6).

Participants 1, 3, 4 and 7 had a history of hay fever, meaning they were likely to have an atopic predisposition. Individuals with this predisposition are at an increased risk of developing atopic dermatitis and often exhibit impaired skin barrier function, including reduced filaggrin expression and increased TEWL (van den Oord et al. 2023; Silverberg 2019). Participants 1, 3 and 4 also showed signs of dry skin, which could have influenced their skin barrier function. However, this was not evident in the baseline TEWL measurements. Only participant 3 showed a slightly elevated TEWL value, which was the highest measurement of the study participants. A high proportion of participants experienced symptoms of hand eczema within the past 12 months or were presented with dry skin at the time of assessment. Hand eczema and dry skin are known to be common among healthcare workers, reflecting the high prevalence of occupational skin problems in this population (van den Oord et al. 2023; Hamnerius et al. 2018).

The study by Arndt and co-workers (2014) reported that they did not find evidence for skin as a significant route of uptake. However, their laboratory set-up was not representative of clinical practice, and they did not measure air concentrations. Their use of EtG is much less specific and sensitive compared with using blood ethanol as the main outcome parameter. Other published exposure studies either did not aim to quantitatively evaluate the contribution from dermal exposure or lacked the controlled, lab-based design needed to draw robust inferences regarding a role for dermal absorption (Miller et al., 2006; Kramer et al. 2007; Kirschner et al. 2007; Hautemanière et al. 2013; Bessonneau et al. 2012; Emerson et al. 2016; Salomone et al. 2018). Both native and skin-only exposures resulted in variability in blood ethanol and blood ethanol-d6 AUC with considerable interindividual differences in the estimated contribution from skin absorption. This interindividual variability may in part be explained by differences in air concentrations of ethanol in the breathing zone of the participant as indicated by the relative standard deviation of ethanol in air during the skin-only conditions (68%) and the native condition (60%). These numbers are in the same range as the interindividual variability in blood ethanol AUC expressed as relative standard deviation: 78% for the skin-only condition and 66% for the native condition. Nevertheless, it is evident that also the skin properties are likely to influence ethanol permeation, as indicated by the observed skin health status (Table 2). Even among participants with skin considered healthy, the condition can vary due to work-related factors such as the intensity of detergent/soap use (in two study participants) and also factors outside work such as mechanical strains in one participant who engaged in bouldering. The impact of person characteristics and lifestyle factors are not sufficiently covered when using workplace standards for air quality. Additional monitoring of internal exposure may be considered for a more accurate assessment of exposure to verify that it is sufficiently controlled and if not, further mitigating actions can be considered. In healthcare practice, this may be done by ethanol biomonitoring as part of an exposure surveillance program (OECD 2022, Hopf, 2025, Schneider et al. 2025).

The results of our laboratory study were used to estimate internal dose with the ConsExpo web application (ConsExpo Web, 2024) to compare these results with the assumption by Hendriks and co-workers (2021) using a dermal absorption of 2% (Hendriks et al., 2021). This resulted in a relative contribution of 81% compared with the integrated uptake from inhalation and dermal absorption. Starting from the scenario for 10 x AHBR per day and using the exact modelling inputs taken from the study by Hendriks and co-workers (2021) we recalculated the dermal absorption fraction required to match the median contribution observed in the current study (43%). Without changing the contribution from inhalation, we obtained a 1.9-fold lower dermal contribution by using an input value of 0.34% for the fixed fraction absorbed (instead of 2%). All other parameters were kept unchanged (see Hendriks et al. and Supplementary material 8 for details).

The current OEL in the Netherlands of 260 mg/m^3^ for a shift of 8 h does not account for dermal absorption. When including the contribution of dermal absorption to total exposure, the current OEL would need to be reduced by 43% to 148 mg/m^3^, to arrive at the same level of protection. In this calculation, we assume that the relative contribution of dermal absorption does not change with the ABHR frequency per shift. However, for 25 ABHR applications, some accumulation is observed for both inhalation and dermal absorption (Fig. 1). Therefore, if frequencies higher than 25 applications per 2 h (or 100 in 8 h) are expected, an additional assessment factor may be needed to account for a potential effect of accumulation. Regarding the variability observed in the contribution of dermal absorption (range 9–87%) the default uncertainty factor of 5 used to cover interindividual variability in workers could be set to a higher level, given the substantial contribution of dermal absorption under the conditions used in the current study.

### Strengths and limitations

The design of a laboratory study was chosen because of the need to control for inhalation exposure. To reduce the contribution of endogenous ethanol formation the study participants were asked to restrict their carbohydrate intake to define a standardized low baseline of blood ethanol at the start, during the exposure and wash out period. To enhance the real-world relevance, healthcare workers were enrolled as study participants. They introduced their routine to apply ABHRs as experienced users, resulting in a realistic assessment of the procedure and presumably greater reproducibility than would be expected with less experienced users. We invited healthcare workers who also routinely use hygienic disinfection products combined with wet-work tasks, including the use of detergent/soap and are familiar glove users. This was supported by a medical exam of the skin during the intake interview. The ABHRs were studied in a series with intermittent glove use, creating periods of skin occlusion that may have contributed to effects on skin barrier function particularly for a polar chemical such as ethanol, which has not been studied under these conditions before (Arndt et al., 2024). Such exposure conditions may influence hydration of the skin and specifically the skin barrier function in both the short and long term which is considered relevant to assess a real-world exposure (Tiedeman et al. 2016; Plum et al. 2020). The interpretation of the results was supported by measurement of TEWL before, during and after the use of the disinfection product. Additionally, ambient temperature, relative humidity and ventilation were recorded in all exposure settings to secure comparability of the two conditions tested.

Repeated blood sampling ethanol analysis was used as primary outcome measure which offers both sensitivity and specificity to generate a concentration-time pattern that could be used to calculate an AUC as the main study finding. This is an improvement relative to earlier attempts to study inhalation and dermal absorption by end-exhaled air analysis (Hautemanière et al., 2013) and urinary EtG excretion (Arndt et al., 2014). To verify that blood ethanol levels could be related to the disinfection product as exogenous exposure, a small amount of deuterated isotope was added. This substance was analysed in blood to be able to confirm its origin from the hand disinfection product and the ABHR procedure as the source.

A limitation is the small sample size and the under-representation of male healthcare workers in the study group. In a way, this reflects the current gender distribution in the target populationof healthcare workers in hospitals in The Netherlands. In follow-up studies with controlled exposures, a larger study sample may allow investigation of host factors to explain interindividual differences in skin type and condition, including the filaggrin genotype as a potential contributor to skin barrier function (Liljedahl et al., 2024).

The study design is relevant to regular ABHR users within the target population and accounts for the use of gloves during patient contact. This is an important consideration, because the resulting periods of occlusion may have a significant impact on dermal absorption of ethanol. Consequently, the study findings should be interpreted with caution, and care should be taken when extrapolating the results to other populations or conditions. The experimental setting also has limitations related to standardising the study design for comparison of our findings with those of follow-up studies. We asked the study participants to follow the ABHR protocol, and their level of physical exercise was probably much lower than during a typical workday in a clinical setting. We did not assess ABHR use without intermittent wearing of gloves, which may be relevant in some healthcare settings. Real-world conditions would probably result in more variability in exposure, which could translate into even higher variability in blood ethanol concentrations both within a person and between individuals.

## Conclusion

High-frequency hygienic disinfection combined with intermittent glove use leads to a significant contribution of dermal absorption of ethanol. Further study of interindividual variability of the contribution of dermal absorption is needed to understand if, and how, the skin barrier changes with frequent use of different types of hand disinfection products (e.g. wet work, soap and solvents) and under occluded conditions (i.e. glove use). In the meantime, occupational health authorities, employers and workers in the healthcare sector should consider additional surveillance by human biomonitoring. Next to blood ethanol, the feasibility of using exhaled air and urineary EtG as potential biomarkers could be explored. A health-based biological limit for such biomarker(s) would then account for the contribution from dermal absorption.

## Supplementary Information

Below is the link to the electronic supplementary material.

Supplementary Material 1
