# Supplementary material for: Contribution of dermal ethanol absorption during laboratory-controlled high-frequency use of alcohol-based hand rubs followed by glove use
Source: Arch Toxicol. 2026 May 13;100(9):3947–60. doi: 10.1007/s00204-026-04441-9 (PMC13453986; doi:10.1007/s00204-026-04441-9)
Supplement: Supplementary file 1 [file 204_2026_4441_MOESM1_ESM.docx]

**Supplementary material**

**Contribution of dermal ethanol absorption during laboratory-controlled high-frequency use of alcohol-based hand rubs followed by glove use**

Paul T.J. Scheepers^a^, Kirsten Lassing^a,b^, Nina C. Wieland^a^, Martien H.F. Graumans^a^, Sander H.P. Driessen^a^, Antzelina Gkintsev^a^, Ioanna Konstantinou^a^, Valérie Leermakers^a^, Hilde Onstek^a^, Maxime Sulter^a^, Lotte van den Oord^c^, Chariya van Dolder^a^, Hendrik Jan Jansen^e^, Emilie M. Hardy^f^, Radu Corneliu Duca^f,g^, Ad Ragas^a^, Frans G.M. Russel^d^, Thomas Rustemeyer^e^

^a^Radboud Institute for Biological and Environmental Sciences, Radboud University Nijmegen, Nijmegen, The Netherlands

^b^Radboudumc Community for Infectious Diseases, Research Institute for Medical Innovation, Department of Medical Microbiology, Radboud University Medical Center, PO Box 1, Nijmegen 6500 HB, The Netherlands

^c^Leiden University Medical Center, Department of Dermatology, Albinusdreef 2, 2333 ZG Leiden, The Netherlands

^d^Department of Pharmacy, Pharmacology and Toxicology, Research Institute for Medical Innovation, Radboud University Medical Center, PO Box 9101, Nijmegen 6500 HB, The Netherlands

^e^Amsterdam University Medical Center, Amsterdam, The Netherlands

^f^Unit Environmental Hygiene and Human Biological Monitoring, Department of Health Protection, Laboratoire national de santé, 3555 Dudelange, Luxembourg

^g^Department Health Protection, Health Directorate, 1445 Strassen, Luxembourg

*Corresponding author: Paul. T.J. Scheepers PhD, Radboud Institute for Biological and Environmental Sciences, Radboud University Nijmegen, Heyendaalseweg 135, 6525AJ Nijmegen, The Netherlands, mail: [paul.scheepers@ru.nl](mailto:paul.scheepers@ru.nl); phone: +31653748401

List of Supplementary materials

1. Inclusion and exclusion criteria
2. Hygienic skin disinfection instructions
3. GC-EI MS parameters and QA/QC data blood ethanol analysis
4. Dietary information
5. Room climate measurements
6. Trans epidermal water loss measurements
7. Blood ethanol and ethanol-d6 concentrations
8. Comparison of the current study with the modelling study by Hendriks et al. (2021)

**Supplementary material 1: inclusion and exclusion criteria**

The participants were recruited from the group of professionally employed users of ABHR in healthcare practice. Because of their employment the study participants were in the working age range (18-67). Participation was restricted to individuals of Caucasian descent because of genetic polymorphisms in the ethanol dehydrogenase (ADH2) and acetaldehyde dehydrogenase gene (ALDH2) see <https://omim.org/entry/100650>. These polymorphisms, which include an inherited deficiency common among Chinese, Korean and Japanese populations (Wall et al, 1997), would substantially increase variability in ethanol as a dosimeter of uptake. Regarding the small group of subjects, we preferred to not introduce this additional source of variability, as it is not relevant to the study objectives and could reduce statistical power. For this reason, we aimed for an even distribution between Caucasian males and females. Other authors have made similar choices when involving small groups of human volunteers (Mitchell et al., 2014).

**Inclusion criteria**

To be eligible to participate in this study, a subject must meet all of the following criteria:

- European descent (Caucasian)
- Employment in healthcare (hospital, GGD or similar)
- Applying >25 ABHR/work shift for at least 3 full shifts/week when at work
- 18-55y

**Exclusion criteria**

A potential subject meeting any of the following criteria was excluded from participation in this study:

- Skin disease^a^
- Liver disease
- Pregnancy
- Lactation

^a^Chronic hand dermatitis was evaluated using a five-point score (Coenraads et al., 2005). Participants with a score of 3 or higher (‘severe’ and ‘very severe’) was excluded.

**References**

Mitchell MC Jr, Teigen EL, Ramchandani VA. (2014) Absorption and peak blood alcohol concentration after drinking beer, wine, or spirits. Alcohol Clin Exp Res. 38(5):1200-4. <https://doi.org/10.1111/acer.12355>.

Wall TL, Peterson CM, Peterson KP, Johnson ML, Thomasson HR, Cole M, Ehlers CL (1997) Alcohol metabolism in Asian-American men with genetic polymorphisms of aldehyde dehydrogenase. Ann Intern Med. 127(5):376-9. <https://doi.org/10.7326/0003-4819-127-5-199709010-00007>.

**Supplementary material 2 – Hygienic hand disinfection instruction** (see reference below)

Hand disinfection should be carried out as follows:

1. Apply hand alcohol from the dispenser to dry hands, without touching the dispenser nozzle.
2. Use enough hand alcohol to fill the well of one hand. This amount is necessary to keep the hands wet for the entire contact time of the hand alcohol (30 seconds).
3. Now rub your hands thoroughly together for about 30 seconds until they are dry. The fingertips, thumbs, the areas between the fingers, and the wrists should also be thoroughly rubbed with the hand alcohol.
4. If the alcohol does not dry or does not dry sufficiently, the effect is reduced and there is also a risk of skin irritation if gloves are subsequently used. Note: Certain parts of the hands are often overlooked. Frequently overlooked parts of the hands are the fingertips, the areas between the fingers, and the thumbs.

**Figure S2a**. Instruction for the procedure in hygienic hand disinfection

**Reference**

Workgroup Infection prevention of the National Institute of Public Health and the Environment (2012) <https://www.dokterhoe.nl/fileadmin/user_upload/documents/gevaarlijke_stoffen/WIP_richtlijn_handhyg.pdf> Accessed 22-12-2025

**Figure S2b.** Laboratory with fume hoods.

**Figure 2b.** The laboratory is arranged so that the researcher and participant work in separate fume hoods (above). The study participant performs the ABHR procedure within their designated fume hood.

**Figure S2c.** Office with arrangement for blood collection and desk for participant.

**Supplementary material 3 – GC-EI MS parameters and QA/QC data blood ethanol analysis**

**Table 3.1.** GC-EI-MS parameters used for the ethanol and ethanol-D6 determination

| **Compound** | **RT (min)** | **Quantifier** | **Qualifier 1** | **LOQ** |
| --- | --- | --- | --- | --- |
|  |  |  | ***(ratio %)*** | **(mg/L)** |
| Ethanol-D_6_ | 1.17 | 33 | 49.0 *(48)* | 0.1 |
| Ethanol | 1.19 | 31 | 45.0 *(65)* | 0.1 |

**Table 3.2a.** QA/QC data blood ethanol concentration analysis with internal standard en serum standard and sample

| **Sample code** | **Type** | **Acq. Date-Time** | **Concentration**  **(mg/L)** | **Final Concentration (mg/L)** | **Accuracy (%)** |
| --- | --- | --- | --- | --- | --- |
| Ctrl_serum_500mgL_1 | Serum standard | 15/06/2023 16:29 | 500 | 501.72 | 100 |
| Ctrl_serum_500mgL_2 | Serum standard | 15/06/2023 18:50 | 500 | 506.50 | 101 |
| Ctrl_WB_300mgL_1 | Sample | 03/07/2023 13:37 | 304 | 351.33 | 116 |
| Ctrl_WB_300mgL_2 | Sample | 03/07/2023 15:30 | 304 | 351.30 | 116 |
| Ctrl_WB_300mgL_3 | Sample | 03/07/2023 17:23 | 304 | 363.77 | 120 |
| Ctrl_WB_300mgL_4 | Sample | 03/07/2023 19:16 | 304 | 366.73 | 121 |
| Ctrl_WB_300mgL_5 | Sample | 03/07/2023 20:59 | 304 | 374.10 | 123 |

**Table 3.2b.** QA/QC data blood ethanol concentration analysis without internal standard in whole blood.

| **Sample code** | **Acq. Date-Time** | **Concentration (mg/L)** | **Final Concentration (mg/L)** | **Accuracy (%)** |
| --- | --- | --- | --- | --- |
| Ctrl_WB_300mgL_1 | 03/07/2023 13:37 | 304 | 321.76 | 106 |
| Ctrl_WB_300mgL_2 | 03/07/2023 15:30 | 304 | 314.87 | 104 |
| Ctrl_WB_300mgL_3 | 03/07/2023 17:23 | 304 | 308.89 | 102 |
| Ctrl_WB_300mgL_4 | 03/07/2023 19:16 | 304 | 313.63 | 103 |
| Ctrl_WB_300mgL_5 | 03/07/2023 20:59 | 304 | 311.89 | 103 |
| Ctrl_WB_304mgL_1 | 23/07/2025 15:17 | 304 | 313.35 | 103 |
| Ctrl_WB_304mgL_2 | 23/07/2025 17:57 | 304 | 299.90 | 99 |
| Ctrl_WB_304mgL_3 | 23/07/2025 20:46 | 304 | 303.55 | 100 |
| Ctrl_WB_304mgL_4 | 23/07/2025 23:26 | 304 | 289.97 | 95 |
| Ctrl_WB_304mgL_5 | 24/07/2025 02:16 | 304 | 273.57 | 90 |
| Ctrl_WB_304mgL_1 | 24/07/2025 12:51 | 304 | 313.63 | 103 |
| Ctrl_WB_304mgL_2 | 24/07/2025 15:09 | 304 | 304.58 | 100 |
| Ctrl_WB_304mgL_3 | 24/07/2025 17:59 | 304 | 316.95 | 104 |
| Ctrl_WB_304mgL_4 | 24/07/2025 20:39 | 304 | 321.37 | 106 |
| Ctrl_WB_304mgL_5 | 24/07/2025 23:19 | 304 | 338.72 | 111 |
| Ctrl_WB_304mgL_6 | 25/07/2025 01:59 | 304 | 341.26 | 112 |
| Ctrl_WB_304mgL_7 | 25/07/2025 04:39 | 304 | 348.25 | 115 |
| Ctrl_WB_304mgL_8 | 25/07/2025 07:19 | 304 | 363.39 | 120 |

**Supplementary material 4 – dietary information**

**Table S3.** Total carbohydrate and sugar intake during the two visits and the 24-hour period preceding each visit is presented in grams (carbohydrates and sugars). For study participant 7 dietary information was not available.

| **Total carbohydrates (sugar) in grams** | **1** | **2** | **3** | **4** | **5** | **6** | **AM (SD)** |
| --- | --- | --- | --- | --- | --- | --- | --- |
| 24h prior to visit 1 | 49.6 (33.3) | 87.4 (22.7) | 52.6 (42.6) | 53.6 (33.8) | 40.1 (24.3) | 60.3 9 (17.8) | 57.3 (29.1) |
| Visit 1 | 32.1 (13.0) | -* | 59.6 (54.3) | 46.9 (30.6) | 29.8 (24.8) | 27.8 (20.7) | 39.2 (28.7) |
| 24h prior to visit 2 | 56.3 (47.4) | 44.0 (35.1) | 46.9 (36.2) | 48.6 (34.1) | 50.7 (38.6) | 31.7 (19.1) | 46.4 (35.1) |
| Visit 2 | 63.7 (48.8) | -* | 54.0 (45.7) | 35.5 (22.0) | 40.1 (33.9) | 27.8 (20.7) | 44.2 (34.2) |
| **Average carbohydrates** | **50 (36)** | **66 (29)** | **52 (45)** | **46 (30)** | **33 (24)** | **37 (20)** | **47 (30)** |

*Data for participant 2 is not reported for the day before the first and second visit.

**Supplementary material 5 – Room climate measurements**

**Table S5.** Temperature, relative humidity and CO2 for both the skin-only and native conditions in the laboratory (exposure) and office (wash-out). For participant 7 climate measurements were not available.

| **Study pariticipant number** | **Condition** | **Temperature** | | **Relative humidity** | | **CO_2_** | |
| --- | --- | --- | --- | --- | --- | --- | --- |
|  |  | **Laboratory** | **Office** | **Laboratory** | **Office** | **Laboratory** | **Office** |
| **1** | Skin-only | 22.2 ± 1.1 | 24.4 ± 0.6 | 72.8 ± 3.8 | 72.8 ± 3.8 | 464 ± 41 | 464 ± 41 |
|  | Native | 21.2 ± 0.1 | 22.2 ± 0.6 | 60.3 ± 1.7 | 49.6 ± 2.0 | 434 ± 27 | 419 ± 22 |
| **2** | Skin-only | 21.0 ± 02 | 22.2 ± 0.4 | 59.6 ± 1.8 | 52.2 ± 1.4 | 383 ± 19 | 394 ± 20 |
|  | Native | 21.3 ± 0.1 | 23.7 ± 0.4 | 76.5 ± 1.1 | 57.4 ± 3.5 | 382 ± 16 | 405 ± 17 |
| **3** | Skin-only | 21.1 ± 01 | 21.1 ± 0.5 | 58.7 ± 1.6 | 52.6 ± 2.6 | 380 ± 16 | 394 ± 20 |
|  | Native | 21.9 ± 1.5 | 24.1 ± 1.1 | 56.9 ± 4.1 | 45.0 ± 3.2 | 413 ± 32 | 441 ± 45 |
| **4** | Skin-only | 19.9 ± 0.7 | 21.0 ± 0.5 | 46.5 ±1.4 | 43.8 ± 1.1 | 421 ± 14 | 457 ± 40 |
|  | Native | 20.1 ± 0.2 | 21.2 ± 1.5 | 37.0 ± 1.8 | 33.1 ± 3.0 | 425 ± 48 | 591 ± 55 |
| **5** | Skin-only | 20.4 ± 1.5 | 23.8 ± 0.1 | 33.0 ± 2.9 | 26.3 ± 0.5 | 413 ± 66 | 385 ± 39 |
|  | Native | 18.8 ± 0.4 | -^a^ | 22.0 ± 0.7 | -^a^ | 487 ± 73 | -^a^ |
| **6** | Skin-only | 20.6 ± 0.5 | 21.5 ± 1.4 | 46.4 ± 0.6 | 44.6 ± 2.7 | 394 ± 62.8 | 411 ± 56 |
|  | Native | 19.5 ± 0.2 | 19.9 ± 0.9 | 44.4 ± 0.6 | 44.3 ± 1.6 | 436 ± 10 | 482 ± 84 |
| **AM ± SD^b^** | **Skin-only** | **20.9 ± 1.1** | **22.3 ± 1.6** | **53 ± 18** | **49 ± 8** | **409 ± 31** | **418 ± 67** |
| **AM ± SD^b^** | **Native** | **20.5 ± 0.4** | **22.2 ± 1.0** | **50 ± 9** | **46 ± 9** | **430 ± 15** | **470 ± 24** |

^a^ Data incomplete due to memory capacity overload; ^b^ Arithmetic mean and standard deviations for study group.

**Supplementary material 6 – Trans epidermal water loss measurements**

**Table 6.1.** TEWL values (g.m^-2^.h^-1^) measured in triplicate (1, 2, 3) with arithmetic means of triplicate and arithmetic means relative to baseline TEWL at t=0 (expressed as % of mean at baseline) on the palmar as well as the dorsal side of the dominant hand of each study participant during the skin only condition (v1) and native condition (V2). For study participant 7 TEWL measurements are not available.

**Figure S5.** The development of TEWL was assessed from baseline at t= 0 (taken as the reference), three measurements during the ABHR series, and the last time point (t=240), which corresponded to the wash-out phase (each point is the average of a measurement in triplicate). For study participant 7, TEWL measurement were not available. v1 refers to the skin only condition and v2 to the native condition.

**Supplementary material 7 – Blood ethanol and ethanol-d6 concentrations**

Time concentration patterns for ethanol (participants 1-7). For ethanol-d6 only results are shown if sufficient blood concentrations were > LOQ (participants 0. 3 and 6).

Participant-1 EtOH EtOH in air: not available AUC skin-only / AUC skin+ inhalation = 28%

Ethanol in blood (mg/L)

| Time (min) | Skin only | Skin + inhalation |
| --- | --- | --- |
| 0 | 0 | 0 |
| 10 | 0.005 | 7.64999 |
| 20 | 0.005 | 21.2263 |
| 30 | 9.49479 | 22.69 |
| 45 | 17.5703 | 24.1393 |
| 60 | 18.4541 | 41.5808 |
| 75 | 0.102102 | 26.6101 |
| 90 | 0.29169 | 19.8708 |
| 120 | 0.005 | 0.07 |
| 150 | 0 | 0.005 |
| 180 | 0.005 | 0.02 |
| 210 | 0.005 | 0.01 |
| 240 | 0.005 | 0 |

**Figure S1a.** Concentration time pattern of ethanol (EtOH) and ethanol-d6 (EtOH-d6) in blood (mg/L) after 25 AHBRs for skin-only exposure (red dotted curve) and for skin and inhalation exposure (blue curve). EtOH in air = time-weighted average concentration of ethanol in air (µg/m^3^); AUC = area under curve for ethanol concentration in blood (mg/L * min).

Participant-1 EtOH-d6 (1% added to ABHR product used) EtOH in air: not available AUC skin-only / skin+ inhalation = 34%

Ethanol in blood (mg/L)

| Time from start exposure (min) | Skin only | Skin + inhalation |
| --- | --- | --- |
| 0 | 0 | 0 |
| 10 | 0.005 | 0.153668 |
| 20 | 0 | 0.309501 |
| 30 | 0.176581 | 0.36 |
| 45 | 0.258509 | 0.351989 |
| 60 | 0.282004 | 0.578105 |
| 75 | 0.005 | 0.394112 |
| 90 | 0 | 0.313216 |
| 120 | 0.005 | 0.005 |
| 150 | 0.005 | 0.005 |
| 180 | 0.005 | 0.005 |
| 210 | 0.005 | 0.005 |
| 240 | 0.005 | 0.005 |

Participant-2 EtOH EtOH in air during exposure: 11 and 208 mg/m3 and during wash-out: 11 and 5 mg/m3) AUC skin-only / AUC skin+inhal = 87%

|  | Skin only | Skin + inhalation |
| --- | --- | --- |
| 0 | 0 | 0 |
| 10 | 0 | 0 |
| 20 | 0 | 0 |
| 30 | 0 | 0 |
| 45 | 0 | 0.03 |
| 60 | 0.04 | 0.553887 |
| 75 | 0.342738 | 0 |
| 90 | 0.07 | 0.104945 |
| 120 | 4.54584 | 0.242088 |
| 150 | 5.47484 | 2.12091 |
| 180 | 7.48703 | 17.7096 |
| 210 | 14.4983 | 9.35005 |
| 240 | 10.1678 | 2.55921 |

**Figure S1b.** Concentration time pattern for ethanol in blood (EtOH) for skin-only exposure (red dotted curve) and for skin and inhalation exposure (blue curve) ) in blood mg/L) after 25 AHBRs. EtOH in air = time-weighted average concentration of ethanol in air (µg/m^3^); AUC = area under curve for ethanol concentration in blood (mg/L * min).

Participant-3 EtOH EtOH in air during expsure: 5 and 63 mg/m3 and during wash-out: 7 and 6 mg/m3 AUC skin-only / AUC skin+inhal = 73%

|  | Skin only | Skin + inhalation |
| --- | --- | --- |
| 0 | 0 | 0 |
| 10 | 0 | 0 |
| 20 | 0 | 0 |
| 30 | 0.1435 | 1.47083 |
| 45 | 0.117228 | 2.25503 |
| 60 | 0.591901 | 11.0377 |
| 75 | 2.28576 | 8.2285 |
| 90 | 0.611569 | 13.2584 |
| 120 | 7.54562 | 22.3366 |
| 150 | 19.896 | 21.4753 |
| 180 | 22.4952 | 18.2724 |
| 210 | 11.5326 | 4.85663 |
| 240 | 6.28788 | 5.65036 |

**F****igure S1c.** Concentration time pattern for ethanol in blood (EtOH) for skin-only exposure (red dotted curve) and for skin and inhalation exposure (blue curve) in blood (mg/L) after 25 AHBRs. EtOH in air = time-weighted average concentration of ethanol in air (µg/m^3^); AUC = area under curve for ethanol concentration in blood (mg/L * min).

Participant-4 EtOH EtOH in air during exposure: 8 and 58 mg/m3) and during wash-out: 6 and 4 mg/m3 AUC skin-only / AUC skin+inhal = 66%

|  | Skin only | Skin + inhalation |
| --- | --- | --- |
| 0 | 0 | 0.06 |
| 10 | 0 | 0.08 |
| 20 | 0 | 0 |
| 30 | 0 | 18.08 |
| 45 | 0 | 28.1531 |
| 60 | 0 | 31.0222 |
| 75 | 12.625 | 12.2345 |
| 90 | 0 | 30.2791 |
| 120 | 0.05 | 53.6796 |
| 150 | 66.2547 | 13.7645 |
| 180 | 21.2902 | 36.2871 |
| 210 | 18.1523 | 0 |
| 240 | 1.25114 | 0 |

Participant-4 EtOH-d6 (1% added to ABHR product used) AUC skin-only / AUC skin+inhal = 61%

|  | Skin only | Skin + inhalation |
| --- | --- | --- |
| 0 | 0 | 0 |
| 10 | 0 | 0 |
| 20 | 0 | 0 |
| 30 | 0 | 0.07 |
| 45 | 0 | 0.200655 |
| 60 | 0 | 0.248984 |
| 75 | 0 | 0 |
| 90 | 0 | 0.207434 |
| 120 | 0 | 0.530024 |
| 150 | 0.650621 | 0 |
| 180 | 0.05 | 0.268325 |
| 210 | 0.035 | 0 |
| 240 | 0 | 0 |

**Figure S1d.** Concentration time pattern for ethanol (EtOH. upper) and ethanol-d6 (EtOH-d6. lower) in blood (mg/L) after 25 AHBRs for skin-only exposure (red dotted curve) and for skin and inhalation exposure (blue curve). EtOH in air = time-weighted average concentration of ethanol in air (µg/m^3^); AUC = area under curve for ethanol concentration in blood (mg/L * min).

Participant-5 EtOH EtOH in air during exposure: 6 and 215 mg/m3 and during wash-out: 6 and 5 mg/m3) AUC skin-only / AUC skin+inhal = 11%

|  | Skin + Inhalation | Skin only |
| --- | --- | --- |
| 0 | 0 | 0.05 |
| 10 | 0 | 0.05 |
| 20 | 0.210555 | 0 |
| 30 | 0 | 0 |
| 45 | 2.62272 | 0.06 |
| 60 | 5.11845 | 0.73694 |
| 75 | 5.30228 | 2.82622 |
| 90 | 13.547 | 2.91244 |
| 120 | 18.9245 | 0.245531 |
| 150 | 20.5317 | 1.87266 |
| 180 | 53.682 | 3.29714 |
| 210 | 22.6404 | 0 |
| 240 | 46.1776 | 16.5329 |

**Figure S1e.** Concentration time pattern for ethanol in blood (mg/L) after 25 AHBRs for skin-only exposure (red dotted curve) and for skin and inhalation exposure (blue curve). EtOH in air = time-weighted average concentration of ethanol in air (µg/m^3^); AUC = area under curve for ethanol concentration in blood (mg/L * min).

Participant-6 EtOH EtOH in air during exposure: 4 and 37 mg/m3 and during wash-out: 0.8 and 0.9 mg/m3 AUC skin-only / AUC skin+inhal = 9 %

|  | Skin only (2nd attempt) | Skin + inhalation |
| --- | --- | --- |
| 0 | 0 | 0 |
| 10 | 0 | 0.08 |
| 20 | 0 | 0 |
| 30 | 0.07 | 0.07 |
| 45 | 0 | 0.05 |
| 60 | 0.100816 | 0.618012 |
| 75 | 0.78748 | 0.07 |
| 90 | 0 | 3.10662 |
| 120 | 0.867079 | 16.3394 |
| 150 | 3.28942 | 7.15098 |
| 180 | 0.06 | 13.7982 |
| 210 | 0.146391 | 12.7066 |
| 240 | 0 | 0.06 |

**Figure S1f.** Concentration time pattern for ethanol in blood (mg/L) after 25 AHBRs L for skin-only exposure (red dotted curve) and for skin and inhalation exposure (blue curve). EtOH in air = time-weighted average concentration of ethanol in air (µg/m^3^); AUC = area under curve for ethanol concentration in blood (mg/L x min).

Participant-7 EtOH EtOH in air during exposure: 2 and 151 mg/m3 and during wash-out 0.8 and 11 mg/m3 AUC skin-only / AUC skin+inhal = 27%

|  | Skin only | Skin + inhalation |
| --- | --- | --- |
| 0 | 0.04 | 0 |
| 10 | 0.05 | 0.06 |
| 20 | 0.606105 | 0.05 |
| 30 | 0.05 | 0.106356 |
| 45 | 10.1434 | 20.9369 |
| 60 | 6.91434 | 8.25257 |
| 75 | 0.09 | 61.2536 |
| 90 | 0.05 | 38.354 |
| 120 | 9.24283 | 24.214 |
| 150 | 56.9843 | 43.3988 |
| 180 | 0.05 | 93.0646 |
| 210 | 0 | 22.4711 |
| 240 | 0.167969 | 33.3428 |

Participant-7 EtOH-d6 (1% added to ABHR product used) AUC skin-only / AUC skin+inhal = 23%

|  | Skin only | Skin + inhalation |
| --- | --- | --- |
| 0 | 0 | 0 |
| 10 | 0 | 0 |
| 20 | 0 | 0 |
| 30 | 0 | 0 |
| 45 | 0 | 0.06 |
| 60 | 0 | 0 |
| 75 | 0 | 0.561766 |
| 90 | 0 | 0.295776 |
| 120 | 0 | 0.155415 |
| 150 | 0.56055 | 0.399462 |
| 180 | 0 | 1.04094 |
| 210 | 0 | 0.137036 |
| 240 | 0 | 0.281799 |

**Figure S1g.** Concentration time pattern for ethanol (EtOH) and ethanol-d6 (EtOH-d6) in blood (mg/L) after 25 AHBRs for skin-only exposure (red dotted curve) and for skin and inhalation exposure (blue curve). EtOH in air = time-weighted average concentration of ethanol in air (µg/m^3^); AUC = area under curve for ethanol concentration in blood (mg/L x min).

**Table S8.** Output from ConsExpo Web modelling based on Hendriks and co-workers (2021) (see appendix 1 for used input) assuming 2% dermal absorption of ethanol (A) and recalculation to estimate the comparative skin absorption % (B) based on the relative contribution of dermal to total uptake of 43% observed in the current study.

| **Modelling Parameters** | **Unit** | **A** | **B** | **Fold change** |
| --- | --- | --- | --- | --- |
| AHBR frequency | /day | 10 | 10 | 1 |
| Dermal absorption (fixed) | % | 2 | 0.34^a^ | 5.9 |
| **Inhalation** | | | | |
| Inhalation mean event concentration | mg/m^3^ | 7200 | 7200 | 1 |
| Peak concentration (TWA 15 min) | mg/m^3^ | 7200 | 7200 | 1 |
| Mean concentration on day of exposure | mg/m^3^ | 5 | 5 | 1 |
| Year average conentration | mg/m^3^ | 5 | 5 | 1 |
| External event dose | mg/kg bw | 2.5 | 2.5 | 1 |
| External dose on day of exposure | mg/kg bw | 2.5 | 2.5 | 1 |
| Internal event does | mg/kg bw | 1.5 | 1.5 | 1 |
| Internal dose on day of exposure | mg/kg bw/day | 1.5 | 1.5 | 1 |
| Internal year average dose | mg/kg bw/day | 1.5 | 1.5 | 1 |
| **Dermal** | | | | |
| Dermal load | mg/cm^2^ | 2.4 | 2.4 | 1 |
| Extenal event dose | mg/kg bw | 33 | 33 | 1 |
| External dose on day of exposure | mg/kg bw | 330 | 330 | 1 |
| Internal event dose | mg/kg bw | 0.66 | 0.11 | 0.17 |
| Internal dose on day of exposure | mg/kg bw/day | 6.6 | 1.15 | 0.17 |
| inernal year average dose | mg/kg bw/day | 6.6 | 1.15 | 0.17 |
| **Integrated** | | | | |
| Internal event dose | mg/kg bw | 0.81 | 0.26 | 0.32 |
| Internal dose on day of exposure | mg/kg bw/day | 8.1 | 2.6 | 0.32 |
| Internal year average dose | mg/kg bw/day | 8.1 | 2.6 | 0.32 |
| **Contribution of dermal route to total uptake** | | | | |
| Internal event does | % | 31% | 7% | 4.4 |
| Internal dose on day of exposure | % | 81% | 43%^v^ | 1.9 |
| Internal year average dose | % | 81% | 43%^v^ | 1.9 |

^a^Estimated by iteration; ^b^Contribution from current lab study (all other input was kept the same).
